# Magnetic resonance imaging offers advantages over computer tomography for measurement of the shoulder stability ratio

**DOI:** 10.1002/jeo2.70423

**Published:** 2025-09-09

**Authors:** Christian D. Schenk, Sebastian Oenning, Jens Wermers, Marcel Wilken, Julia Sußiek, Arne Riegel, Michael J. Raschke, J. Christoph Katthagen

**Affiliations:** ^1^ Department for Trauma‐, Hand‐ and Reconstructive Surgery University Hospital Münster Münster Germany; ^2^ Department of Engineering Physics University of Applied Sciences Münster Germany; ^3^ Department for Radiology University Hospital Münster Münster Germany

**Keywords:** cartilage, CT imaging, glenoid concavity, labrum, MRI imaging, shoulder stability

## Abstract

**Purpose:**

Recent studies indicate that bony shoulder stability ratio (BSSR) is a useful parameter for estimating glenohumeral stability provided by concavity‐compression. In cases of bony glenoid defects, the BSSR may help to optimise surgical decision‐making. However, the shapes of cartilage and labrum differ from the subchondral bony concavity. The aim of this study was to investigate the influence of cartilage and labrum on glenoid concavity and glenohumeral stability.

**Methods:**

Ten cadaveric shoulders were examined by computer tomography (CT) and magnetic resonance imaging (MRI). Thereby radius, depth and stability ratio in anterior‐posterior and superior‐inferior directions were measured. MRI measurements were performed once including and once excluding the labrum. From these, BSSR, osteochondral shoulder stability ratio (OSSR) and fibrocartilaginous shoulder stability ratio (FCSSR) were calculated, and correlations were investigated to assess the transferability between imaging modalities.

**Results:**

CT and MRI did not provide comparable results due to the influence of cartilage and labrum. The FCSSR showed a significantly higher stability ratio than BSSR in anterior‐posterior and the same tendency in superior‐inferior direction. OSSR and BSSR did not differ significantly. The labrum contributed to a significantly higher depth and lower radius in the anterior‐posterior direction. Cartilage alone led to a significantly lower radius in both directions, without significant differences in depth. Comparison of CT and MRI measurements showed only weak correlations.

**Conclusion:**

Labrum and cartilage lead to an increased depth and decreased radius, resulting in a higher glenoid concavity and, consequently, a higher stability ratio. Comparing FCSSR and BSSR, the influence of labrum and cartilage led to a 25% higher stability ratio in anterior‐posterior direction and a 7.7% higher stability ratio in superior‐inferior direction. By finding no significant differences between bony and osteochondral stability ratios, the labrum of the glenoid appears to be the major factor for the increase in stability ratio. Moreover, CT and MRI measurements showed no transferability.

**Level of Evidence:**

Level II, diagnostic study.

**Abbreviations**
3D OSSR: 3 dimensional osteochondral shoulder stability ratio
AP: anterior‐posterior
BSSR: bony shoulder stability ratio
CT: computer tomography
d.f.z.: deviation from zero
FCSSR: fibrocartilaginous shoulder stability ratio
MA: measuring arm
MPR: multiplanar reconstruction
MRI: magnetic resonance imaging
MRI‐e: magnetic resonance imaging—excluding labrum
MRI‐i: magnetic resonance imaging—including labrum
OSSR: osteochondral shoulder stability ratio
PDW: proton density weighted
SD: standard deviation
SI: superior‐inferior
SR: stability ratio

## INTRODUCTION

The glenohumeral joint offers the highest mobility of all human joints. The ball‐and‐socket configuration of the humeral head within the glenoid cavity results in poor guidance of the humerus and a predisposition to glenohumeral dislocation [26]. Approximately 90% of all shoulder dislocations are traumatic anterior dislocations [38, 39]. While most traumatic dislocations involve labrum injuries, about 40%–50% of all first‐time dislocations cause anterior glenoid bone loss [6, 8, 36, 45]. Shoulder stability is physiologically maintained by the principle of concavity‐compression [23, 25]. The rotator cuff's compressive forces, glenohumeral ligaments and joint capsule stabilise the humeral head within the concave‐shaped glenoid [12, 29]. Glenoid cartilage and the labrum increase the glenoid concavity and by that provide additional stability [49, 50].

Glenoid bone loss and consequent loss of concavity is a risk factor for recurrent dislocation [28, 29, 32, 34, 49]. The indications for bony glenoid refixation or bone graft augmentation depend on the bony defect size. The amount of glenoid bone loss leading to recurrent instability was traditionally reported to range between 20 and 25% of the glenoid width [2]. However, recent literature shows that already a bone loss between 7.5% and 17.5% can lead to decreased stability and an increased risk for recurrent instability, indicating that current value of critical bone loss may be lower than traditionally reported [2, 5, 10, 20, 36, 41, 43, 44]. Bathia et al. investigated 12 cadaveric specimens with sequential bone loss by undergoing osteotomy from 10% to 40% of glenoid width and reported glenoid concavity depth and bony shoulder stability ratio (BSSR) undergoing progressive deformation with increasing bone loss. It is important to mention that 90% of this change occurred already with a 10% glenoid defect. Moreover, the traditionally reported threshold of 20%–25% as single criterion for surgical treatment is a matter of discussion [22, 27, 28, 35, 48]. A high rate of recurrent instability after surgical treatment appears to result from other factors that have not been considered yet [3, 37].

In recent biomechanical studies, glenoid concavity of the bony socket was considered as a relevant stabilising factor of the glenohumeral joint [15, 25, 28, 29, 34, 51]. The computer tomography (CT)‐based BSSR was established by Moroder et al. [29] to quantify glenoid concavity by using Pythagorean trigonometric identities resulting in a formula including radius (*r*) and depth (*d*) of the glenoid and is shown in the following:

$$\frac{\sqrt{1-{\left(\frac{r-d}{r}\right)}^{2}}}{\frac{\left(r-d\right)}{r}}.$$

However, by using CT‐based measurements to calculate the BSSR, cartilaginous and labral structures are not taken into account, although having a significant influence on concavity [25, 47]. In consequence, the use of magnetic resonance imaging (MRI) scans could provide a more precise evaluation of the anatomical concavity, including articular cartilage and the labrum.

The aim of this study was to evaluate the influence of labrum and cartilage on glenoid concavity and therefore on shoulder stability ratio by comparing measurements in CT and MRI. Moreover, by investigating correlations between CT‐ and MRI‐derived stability ratios, the transferability of CT to MRI and vice versa was evaluated. It was hypothesised that MRI measurements allow a precise assessment of glenoid concavity, so that tactile measurements in a robotic test setup were performed to validate precision of MRI. Furthermore, it was hypothesised that regarding measurements of radius, the use of a sphere instead of a unidirectional radius would lead to a more realistic approach.

## METHODS

### Study design

To analyse glenoid concavity, CT and MRI scans as well as tactile measurements with a measuring arm were performed in human cadaveric shoulders. In CT and MRI scans, multiplanar reconstruction (MPR) was performed to allow precise plane alignment and concavity measurements. In CT scans, the bony concavity according to the BSSR, established by Moroder et al. [29], was evaluated. In MRI scans, the following two measurements were performed: (a) assessment of the fibrocartilaginous concavity including articular cartilage and the labrum (MRI‐i), and (b) assessment of the osteochondral concavity including articular cartilage but excluding the labrum (MRI‐e). The tactile measurement with the measuring arm was performed as a reference, evaluating the precision of MRI. Summarising all measurements and the resulting stability ratios, four testing stages were defined:

1. CT‐based BSSR (CT → BSSR).
2. MRI (including labrum) based fibrocartilaginous shoulder stability ratio (MRI‐i → FCSSR).
3. MRI (excluding labrum) based osteochondral shoulder stability ratio (MRI‐e → OSSR).
4. Tactile measurement‐based osteochondral shoulder stability ratio (Tactile measurement → 3D‐OSSR).

### Specimen preparation

**Figure 1:**
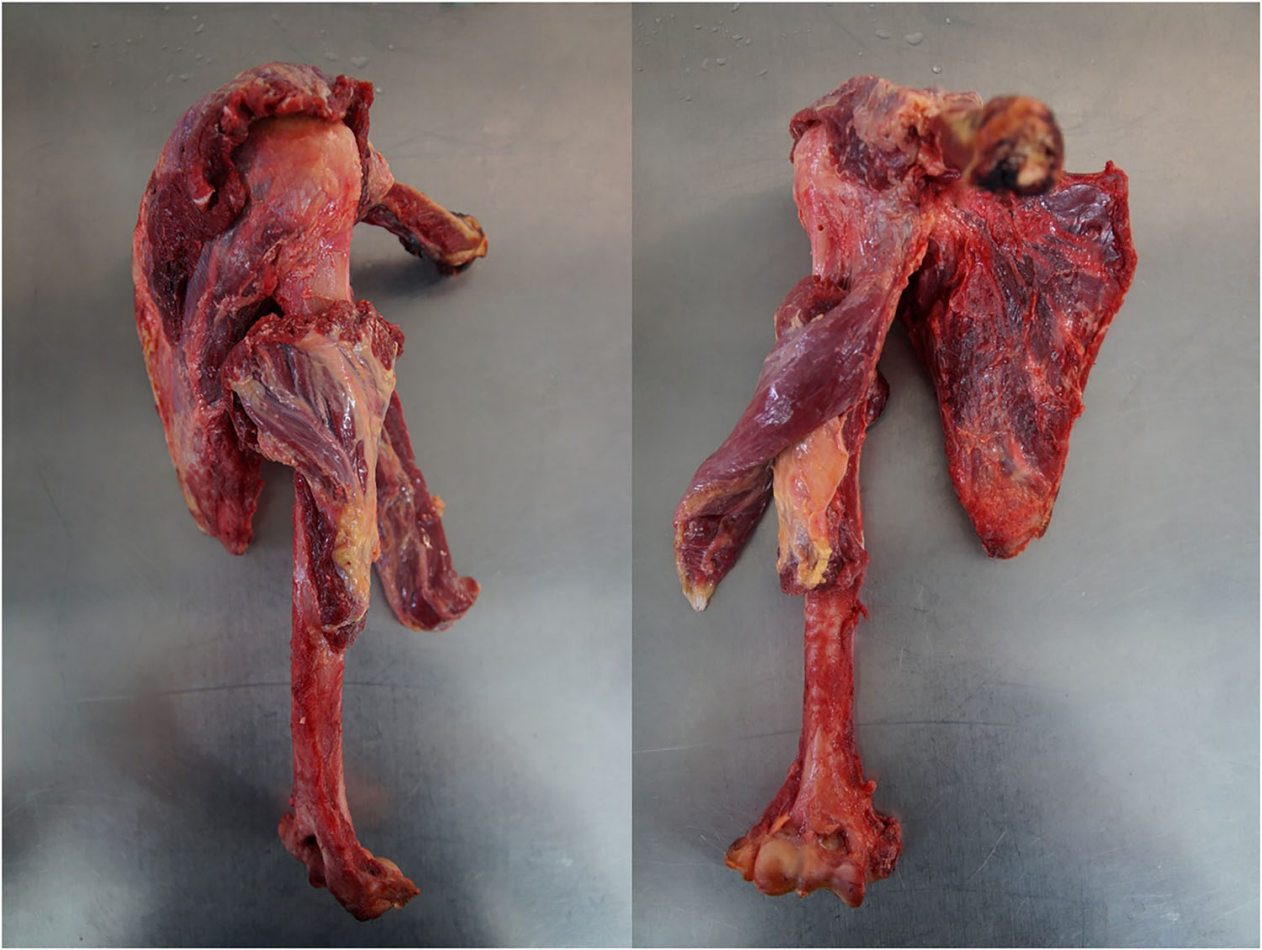
Preparation of shoulder specimen before performing the computer tomography scan. Left: View from posterior side; Right: View from anterior side. Exemplarily, a right shoulder is shown.

For this study, 11 fresh‐frozen, human cadaveric shoulder joints were obtained from the University of Lübeck, Germany. All donors of the human cadaveric specimens provided written consent to use their bodies for scientific and/or educational purposes. The study was approved by the institutional review board (IRB No. 2022‐323‐f‐S, University of Münster, Germany). During specimen preparation, all specimens were carefully examined for macroscopic, osteochondral and labral glenoid lesions. One specimen was excluded from testing due to a posterior fracture of the glenoid. Thus, *n* = 10 specimens with a mean age of 86.9 (±7.66; 76–98) years were included in this study (one right, nine left, three female and seven male). After the specimens were thawed at room temperature, MRI scans were performed prior to soft tissue dissection to create conditions as physiological as possible. After that, the skin, subcutaneous tissue, the biceps and deltoid muscles were removed. The rotator cuff, glenohumeral ligaments, joint capsule and articular cartilage remained intact (see Figure 1). CT scans were then performed in this condition to simulate in vivo conditions as closely as possible.

**Figure 2:**
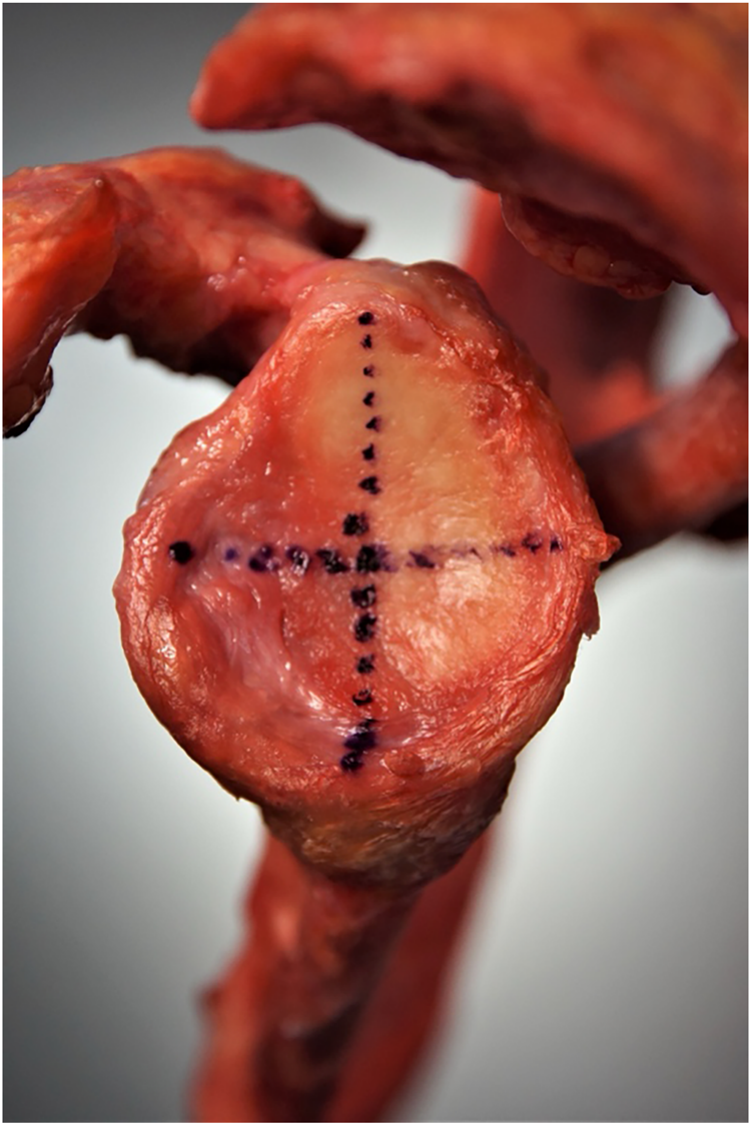
Lateral view of the left glenoid with dissected labrum.

To perform tactile measurements, the articular surface of the glenoid needed to be exposed. Thus, the rotator cuff, glenohumeral ligaments, capsule and labrum, as well as the humerus and the clavicle were removed (see Figure 2).

### CT and MRI‐based measuring methods

**Figure 3:**
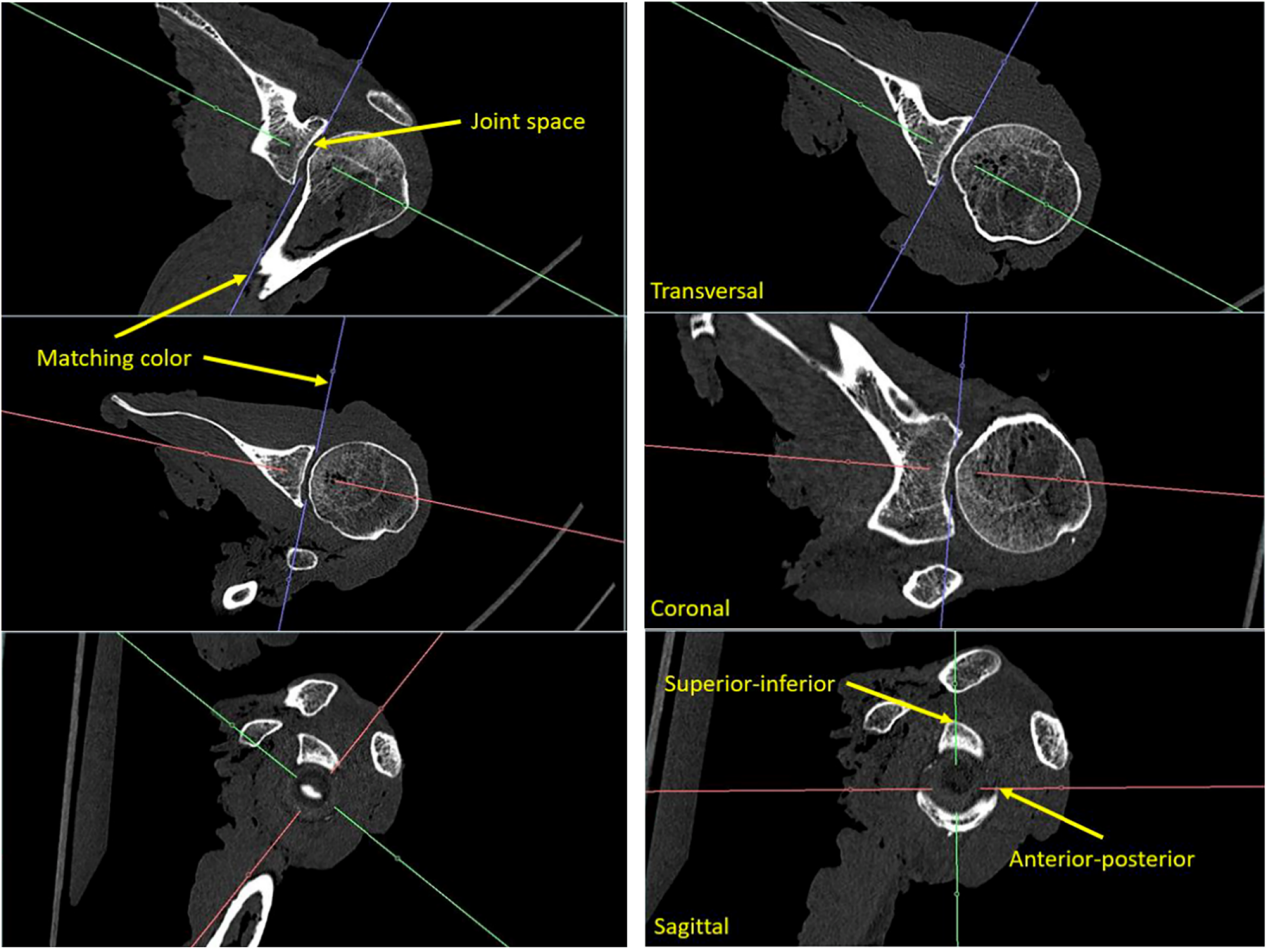
Left**:** Coordinate system of a left shoulder before axes alignment. Right: Coordinate system after axes alignment. In all pictures a computer tomography scan of one of the specimens is used exemplarily.

CT scans (SOMATOM Definition AS) were performed with a slice thickness of 0.6 mm and the bone window was used for measurements. In the MRI (PHILIPS Achieva 3.0 Tesla MRI), T1‐weighted and PDW scans of each specimen were performed with a slice thickness of 0.5 mm. Six external receiver coils were placed around the shoulder to improve imaging quality. The reconstructed isometric voxel size was 0.33 mm. The radiological measurements in MRI and CT scans including individual MPR were performed with Aquarius iNtuition (version 4.4, TeraRecon). Specimen‐specific coordinate systems were aligned to the individual glenoids in both MRI and CT scans, separately. In the CT scans, the superior‐inferior (SI) axis was aligned to the most superior and most inferior point of the bony glenoid. The anterior‐posterior (AP) axis was aligned to the most anterior and posterior point of the glenoid. The mediolateral axis was aligned orthogonally to both priorly defined axes (see Figure 3).

**Figure 4:**
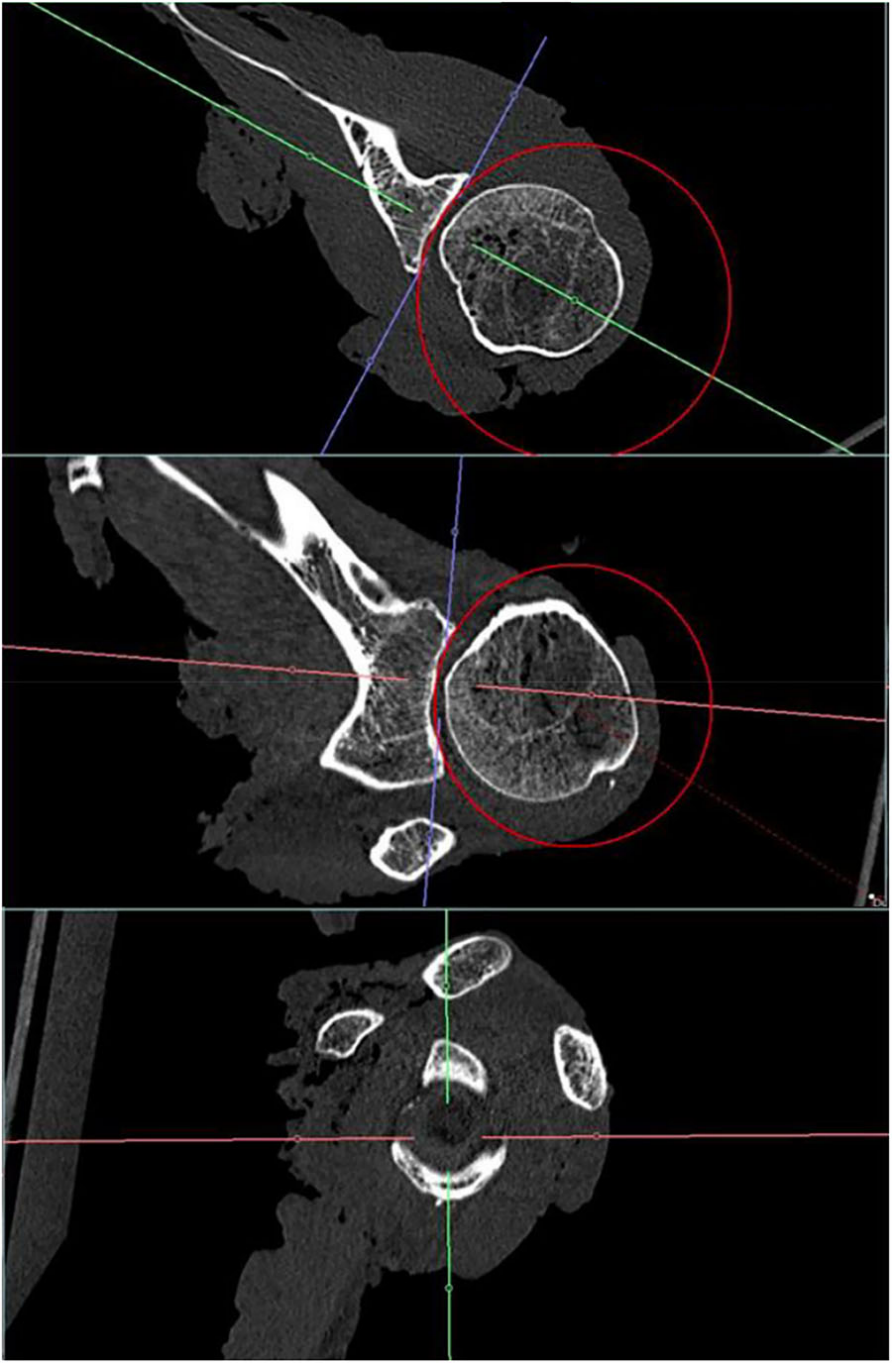
Example of radius measurement in computer tomography, using the best fit circle, matching the curvature of a left glenoid without penetrating the articular surface.

In MRI scans, coordinate system alignment was performed analogously. However, for assessment of the osteochondral concavity, the glenoid articular cartilage was respected to define the glenoid edges. For evaluating fibrocartilaginous concavity, the labrum was additionally included to define the edges of the glenolabral cavity. To assess the SR, glenoid radius and depth were measured. The radius was measured using a best‐fit circle, matching the glenoids' curvature without penetrating the articular surface (see Figure 4). Regarding the glenoids curvature, the BSSR, osteochondral (OSSR) and labral (FCSSR) morphologies were taken into account in CT and MRI scans, respectively.

**Figure 5:**
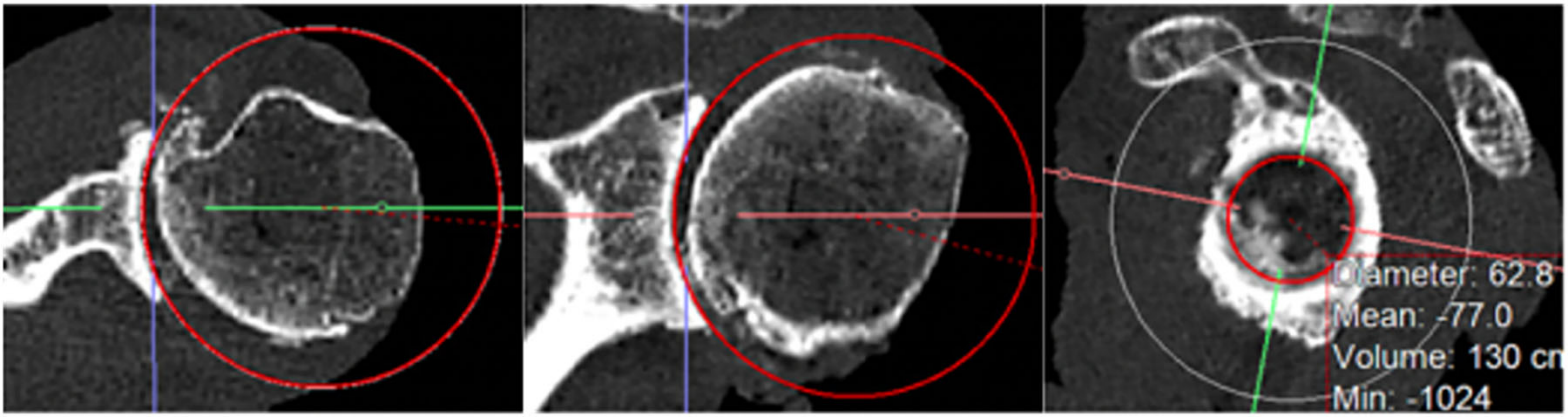
Sphere measurement in computer tomography in transversal (left), coronal (middle) and sagittal (right) planes regarding a left glenoid.

Moreover, by giving a more realistic approach, radius was additionally measured three‐dimensionally by using a sphere. The sphere was created out of a predefined circle in coronal plane, which was defined to be the best plane for creating this first circle, because of having the highest curvature. After creating the sphere, accuracy of fit was confirmed by going through sagittal axis for all possible section views and, if necessary, alignment was corrected to fit the cavity the best way. After that, the centre of the coordinate system was repositioned to the centre of curvature, which was assumed to be the centre of the circular shadow, which can be seen in Figure 5 (right picture). The idea behind using a sphere instead of a directional radius, resulting from one plane, was to get a better representation of the three‐dimensional aspect of the humeral head and to get a more realistic approach.

**Figure 6:**
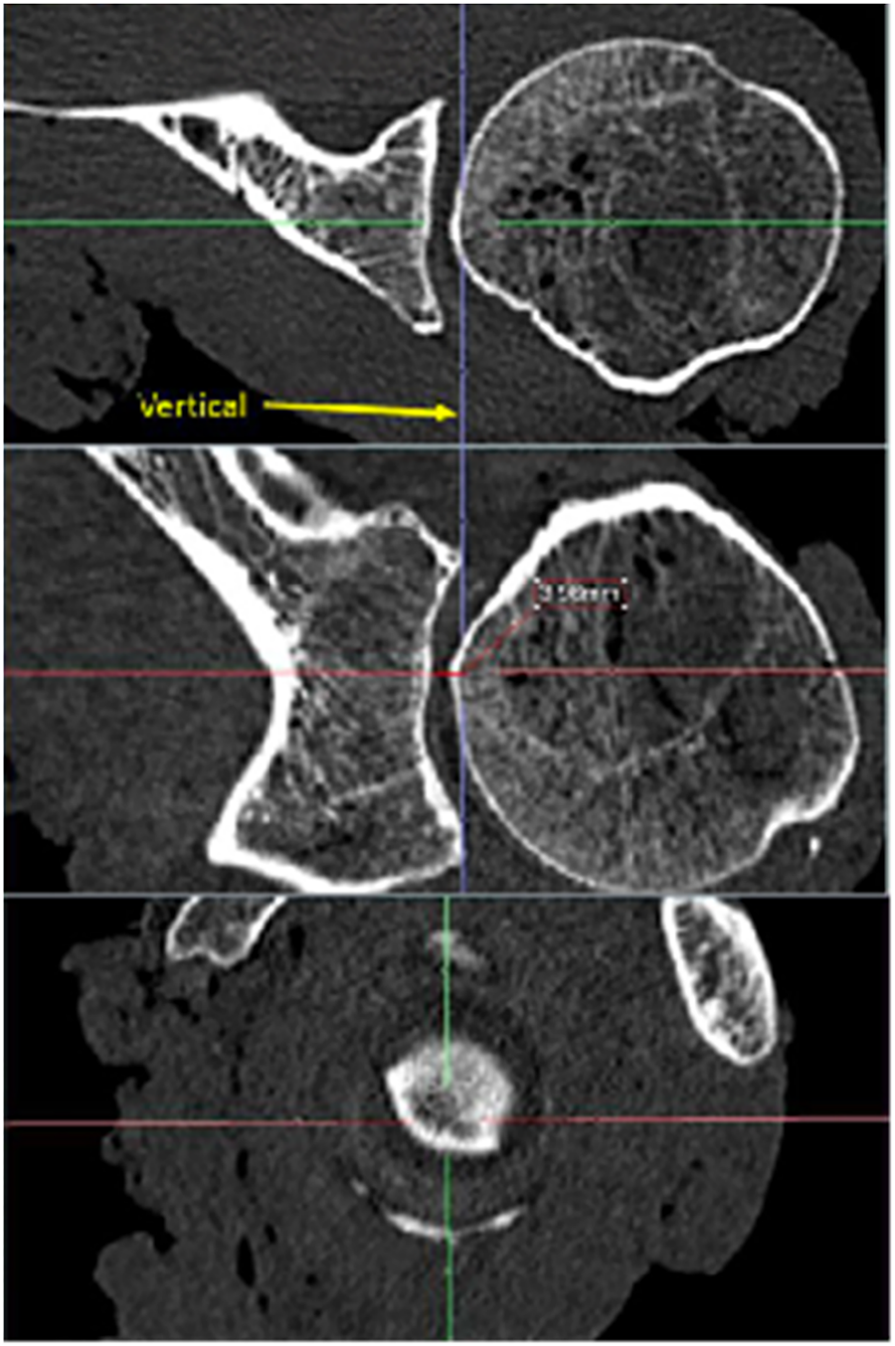
Example of depth measurement in a left glenoid in computer tomography, using a tangent line to the glenoid rim and an orthogonal line in the midpoint of the tangent to the articular surface.

Glenoid depth was measured by aligning a tangent between the most superior and inferior, as well as the most anterior and posterior points of the glenoid rim. Here, the bony (BSSR), osteochondral (OSSR) and fibrocartilaginous (FCSSR) structures were respected. Depth was measured orthogonally from the midpoint of the tangent to the articular surface of the glenoid, while bony and chondral surfaces were used in CT and MRI scans, respectively (see Figure 6).

**Figure 7:**
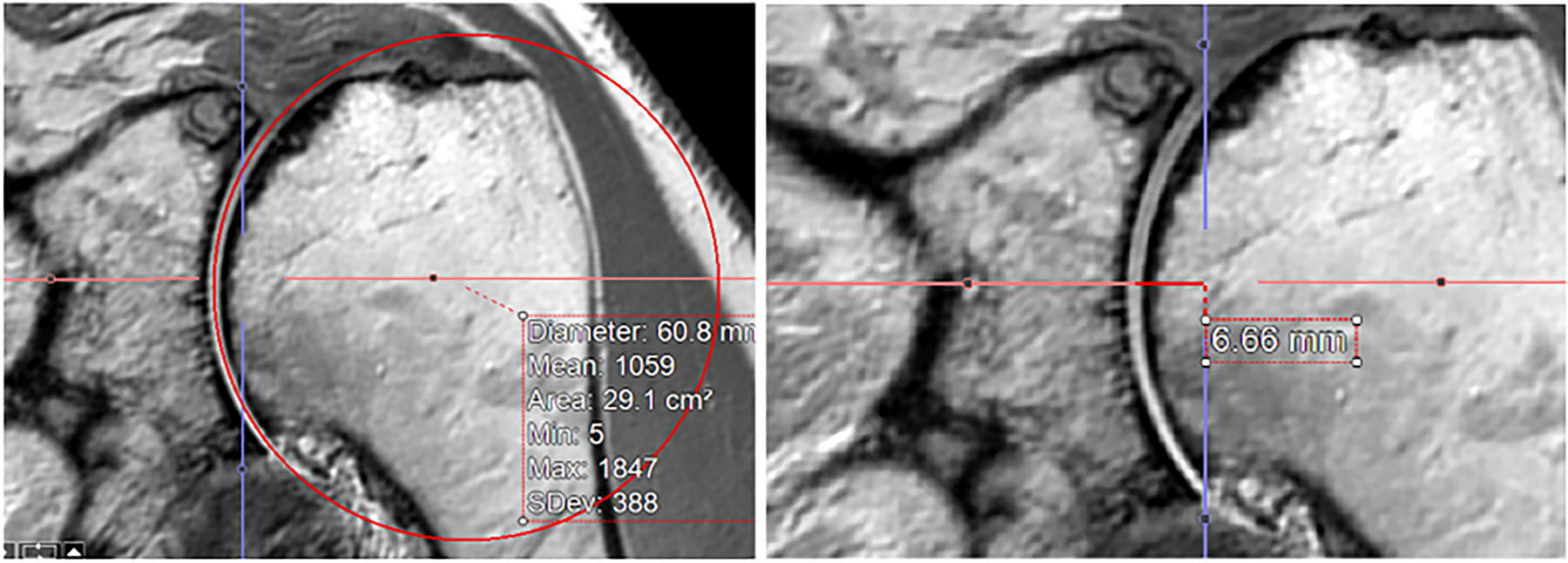
Example of radius and depth measurement in magnetic resonance imaging, considering cartilage and labrum of a left glenoid in superior‐inferior direction. The radius on the left picture was measured using a best‐fit circle, matching the curvature of the glenoid without penetrating the articular surface. The glenoid depth on the right picture was measured by aligning a tangent between the most superior and inferior points of the glenoid rim.

Assessing glenoid radius and depth in both coronal and axial planes, concavity was measured in both SI and AP directions. Visualising the measurements also for MRI a further example for measurement of radius and depth is shown in Figure 7.

### Tactile measurements

**Figure 8:**
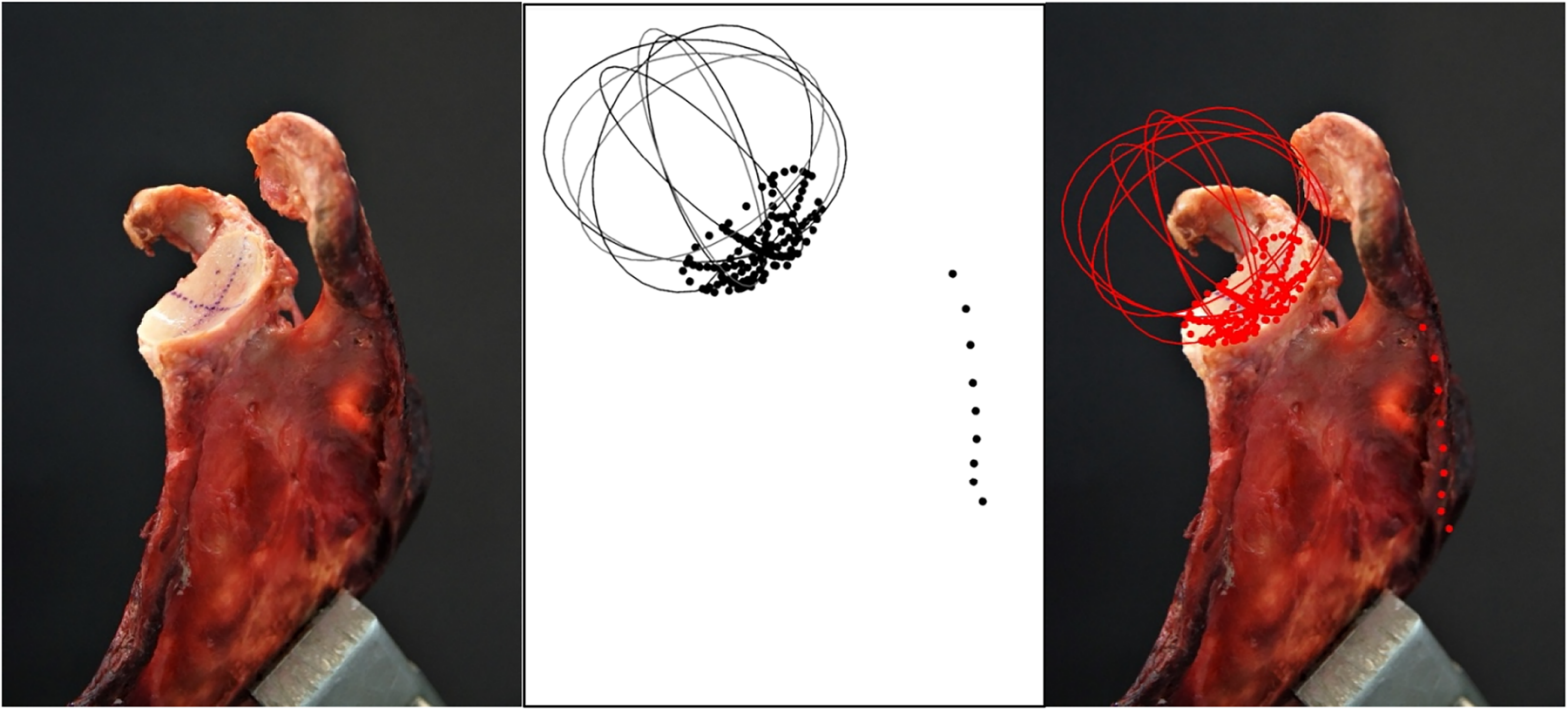
Landmarks measured with the point tip of the measuring arm. Left picture shows a left scapula, fixated in a vice. Picture in the middle shows a screenshot of the software, showing the landmarks (dots) measured with the measuring arm and calculated radii (circles). Right picture shows an overlay of scapula and landmarks to demonstrate fitting. Landmarks are coloured red for better visualisation.

Tactile measurements were performed by putting the scapulae in a vice with the glenoid pointing upward. Because for tactile measurements humeral head, capsule and ligaments had to be removed, a natural positioning of the labrum could not be guaranteed. Thus, the labrum was dissected, as well, and the osteochondral concavity was measured. Measurements were performed with a 3D measuring arm (Absolute Arm 8320‐7, Hexagon Metrology) with a measurement error of <0.05 mm. A standard protocol was created to obtain a high comparability between the measurements. First, 10 reference points were set on both the scapular spine and the inferior angle of the scapula to simplify differentiation between left and right specimens. After that, glenoid landmarks were defined, starting at the centre and continuing with ten reference points in a line to the glenoid rim in anterior direction. Further lines of ten reference points, each, were set in 45° angles, starting at the centre point of the glenoid and splitting the glenoid surface in eighths. In every eighth of the glenoid, six landmarks were added defining three reference points on both, the articular surface and the glenoid rim. The resulting 149 glenoid reference points are shown in Figure 8. The reference points on the glenoid rim were used to generate SI and AP axes creating the same orientation as in MRI scans. In a similar way, the necessary tangents were aligned by the specific software of the measuring arm (Absolute Arm 8320‐7 by HEXAGON) to allow measurements of glenoid radius and depth.

### Statistical evaluation

The measured values are shown as mean, standard deviation and 95% confidence interval. For statistical analyses, paired *t*‐tests were used with a significance level of *p* < 0.05. All results were checked for normal distribution by using the Shapiro–Wilk‐test. For analysing the transferability between CT and MRI, a linear regression model was performed, and the correlation coefficient ‘*r*’ was used. To confirm a postulated correlation the deviation from zero (d.f.z.) was calculated. For documentation, processing and graphical visualisation of all measurements, Microsoft Excel 2016 (Microsoft Corporation) was used. Statistical analyses were performed with GraphPad Prism 9.2 (GraphPad Software).

## RESULTS

### Anterior‐posterior glenoid concavity

**Figure 9:**
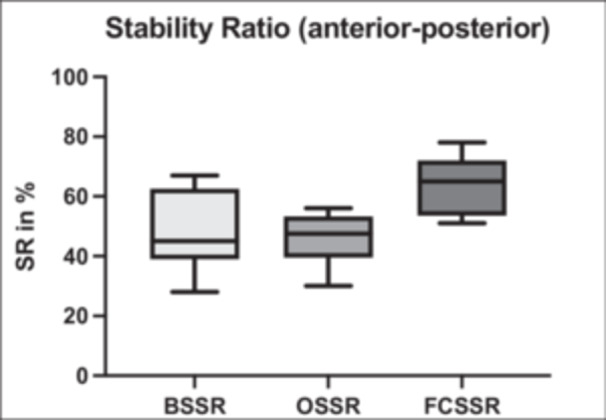
Comparison of stability ratio in anterior‐posterior direction (CT: BSSR, MRI‐e: OSSR, MRI‐i: FCSSR). CT, computer tomography; BSSR, bony shoulder stability ratio; FCSSR, fibrocartilaginous shoulder stability ratio; OSSR, osteochondral shoulder stability ratio.

Assessing the AP glenoid concavity in axial planes, a significantly higher FCSSR, based on the MRI‐i testing stage, was found compared to the CT‐based BSSR (64.00% vs. 48.00%, *p* < 0.01) and compared to the MRI‐e‐based OSSR (64.00% vs. 45.70%, *p* < 0.01). In the linear regression model, the FCSSR showed only weak correlation with the BSSR (*r* = 0.39) and the MRI‐based OSSR (*r* = 0.05). The CT‐based BSSR and MRI‐based OSSR did not differ significantly (48.00% vs. 45.70%, *p* = 0.68). The correlation between BSSR and MRI‐based OSSR was low (*r* = 0.18) and showed no significant deviation from zero (d.f.z.). The measured SR, based on the different measurement modalities, are visualised in Figure 9. Analogously to the increased FCSSR, MRI‐i based glenoid radius was 6.58 mm lower (*p* = 0.02), and glenoid depth was 1.09 mm higher (*p* < 0.01) compared to CT‐based bony measurements. Comparing MRI‐i and MRI‐e, glenoid depth showed 1.86 mm and therefore significantly higher values in the MRI‐i measurements (*p* < 0.001), while radius values were 0.18 mm lower and did not differ statistically significantly (*p* = 0.86). All measurement values are shown in detail in Table 1.

**Table 1:** Comparison of measurement of radius, depth and stability ratio in anterior‐posterior direction in CT, MRI in‐/exclusive labrum and tactile measurement with MA.

	CT	MRI‐i	*p*‐value
Radius (mm)	34.43 ± 6.40	27.86 ± 2.13	**<0.05**
Depth (mm)	3.30 ± 1.25	4.39 ± 1.04	**<0.01**
SR (%)	48.00 ± 13.38	64.00 ± 9.56	**<0.01**

	CT	MRI‐e	*p*‐value
Radius (mm)	34.43 ± 6.40	27.68 ± 2.89	**<0.05**
Depth (mm)	3.30 ± 1.25	2.53 ± 0.79	0.14
SR (%)	48.00 ± 13.38	45.70 ± 8.91	0.68

	MRI‐e	MRI‐i	*p*‐value
Radius (mm)	27.68 ± 2.89	27.86 ± 2.13	0.86
Depth (mm)	2.53 ± 0.79	4.39 ± 1.04	**<0.001**
SR (%)	45.70 ± 8.91	64.00 ± 9.56	**<0.01**

	MA	MRI‐e	*p*‐value
Radius (mm)	30.70 ± 5.10	27.68 ± 2.89	0.14
Depth (mm)	2.14 ± 1.17	2.53 ± 0.79	0.40
SR (%)	39.30 ± 14.06	45.70 ± 8.91	0.24

*Note*: Statistically significant *p*‐values are presented in bold.

Abbreviations: CT, computer tomography; MA, measuring arm; MRI, magnetic resonance imaging; MRI‐e, magnetic resonance imaging—excluding labrum; MRI‐i, magnetic resonance imaging—including labrum; SR, stability ratio.

### Superior‐inferior glenoid concavity

**Figure 10:**
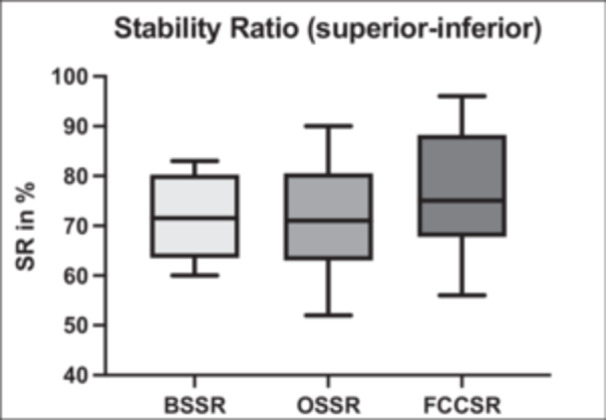
Comparison of stability ratio in superior‐inferior direction (CT: BSSR, MRI‐e: OSSR, MRI‐i: FCSSR). CT, computer tomography; BSSR, bony shoulder stability ratio; FCSSR, fibrocartilaginous shoulder stability ratio; OSSR, osteochondral shoulder stability ratio.

Assessing the SI glenoid concavity in coronal planes, no significant difference was found between FCSSR and BSSR (77.10% vs. 71.20%, *p* = 0.29), or between FCSSR and OSSR (77.10% vs. 71.00%, *p* = 0.12). In the linear regression model, the FCSSR showed only a weak correlation with the BSSR (*r* = 0.19), but a nearly strong correlation with the MRI‐based OSSR (*r* = 0.59). The CT‐based BSSR and MRI‐based OSSR did not differ significantly (71.20% vs. 71.00%, *p* = 0.95). The correlation between BSSR and MRI‐based OSSR was moderate (*r* = 0.47) and showed no significant d.f.z. The measured SR values, based on the different measurement modalities, are visualised in Figure 10. Analogously to the nonsignificant difference between FCSSR and BSSR, there were no significant differences in radius and depth measurements. The MRI‐i based glenoid radius showed the same tendency as in the AP direction and was 3.31 mm lower (*p* = 0.07), and glenoid depth did not differ (*p* = 0.99) compared to the CT‐based bony measurements. Comparing MRI‐i and MRI‐e testing stages, no significant differences in radius (*p* = 0.28) or depth (*p* = 0.06) were found as well. All measurements are shown in detail in Table 2.

**Table 2:** Comparison of measurement of radius, depth and stability ratio in superior‐inferior direction in CT, MRI in‐/exclusive labrum and tactile measurement with measuring arm (MA).

	CT	MRI‐i	*p*‐value
Radius (mm)	31.01 ± 3.54	27.70 ± 2.85	0.07
Depth (mm)	5.66 ± 5.28	5.66 ± 1.10	0.99
SR (%)	71.20 ± 8.34	77.10 ± 13.05	0.29

	CT	MRI‐e	*p*‐value
Radius (mm)	31.01 ± 3.54	27.12 ± 2.04	0.02
Depth (mm)	5.66 ± 5.28	4.99 ± 1.13	0.08
SR (%)	71.20 ± 8.34	71.00 ± 11.50	0.95

	MRI‐e	MRI‐i	*p*‐value
Radius (mm)	27.12 ± 2.04	27.70 ± 2.85	0.28
Depth (mm)	4.99 ± 1.13	5.66 ± 1.10	0.06
SR (%)	71.00 ± 11.50	77.10 ± 13.05	0.12

	MA	MRI‐e	*p*‐value
Radius (mm)	29.62 ± 3.50	27.12 ± 2.04	**<0.01**
Depth (mm)	5.40 ± 0.67	4.99 ± 1.13	0.12
SR (%)	71.30 ± 9.55	71.00 ± 11.50	0.95

*Note*: Statistically significant *p*‐values are presented in bold.

Abbreviations: CT, computer tomography; MA, measuring arm; MRI, magnetic resonance imaging; MRI‐e, magnetic resonance imaging—excluding labrum; MRI‐i, magnetic resonance imaging—including labrum; SR, stability ratio.

### Tactile measurements by measuring arm

**Figure 11:**
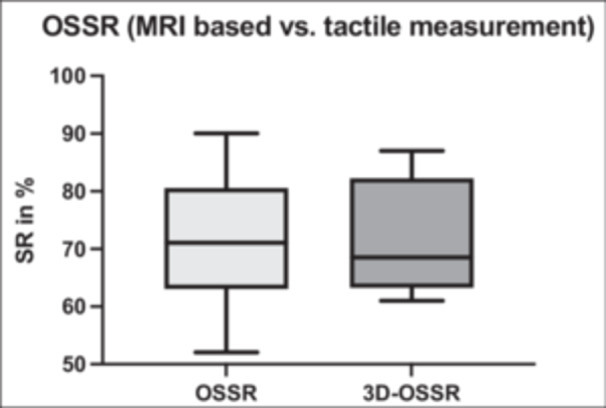
Comparison between magnetic resonance imaging (MRI)‐based osteochondral shoulder stability ratio (OSSR) and tactile measurement‐based shoulder stability ratio (3D‐OSSR).

Comparison of MRI‐based OSSR and 3D‐OSSR, measured in our robotic test setup using the measuring arm, provided significant different values regarding radius (27.12 vs. 29.62 mm, *p* = 0.0023), but no significant difference for depth measurements (4.99 vs. 5.40 mm, *p* = 0.12) and SR (71.00 vs. 71.30, *p* = 0.95). In a linear regression model, strong correlations of *r* = 0.9 were found for radius and *r* = 0.77 for depth, both with significant d.f.z. A comparison of MRI‐based OSSR and tactile measurement‐based 3D‐OSSR is shown in Figure 11.

### Sphere measurements

The goal of this measurement was to determine the lowest radius of a sphere, which fits the whole articular surface of glenoid and to prove, if using a sphere instead of a directional radius is a more realistic approach. If a measured radius in AP or SI would be larger than the other, a humeral head with the radius of the bigger one would not fit to the glenoid. Moreover, it is already known that the humeral head is more ovoid shaped than spherical [13, 18], but while this anatomical knowledge is not yet clearly enough described in literature for leading to precise replicas in specimens we still use a spherical approximation of the humeral head [30, 31]. Sphere radii showed less variation between measurement groups, showing a decrease in standard deviation and a smaller range of values. Concerning the calculated SR, it can be concluded, that using sphere radii instead of directional radii leads to a less deviating approach for estimation of stability. Comparison of measurements of radii are shown additionally in Table 3.

**Table 3:** Comparison of radius measurement in anterior‐posterior and superior‐inferior direction as well as three‐dimensional measurement using a sphere.

Radius (mm)	Mean ± SD	Range
Anterior‐posterior	34.43 ± 6.40	20.80 (26.35–47.15)
Superior‐inferior	31.01 ± 3.54	9.75 (26.35–36.10)
Sphere	27.99 ± 2.70	9.4 (22.6–32.0)

## DISCUSSION

CT and MRI did not provide comparable results due to differences in glenoid surface, caused by cartilage and labrum. Glenoid concavity was therefore strongly influenced by the existence of labrum, while cartilage itself had a minor influence. Summarising these findings, it can be concluded that a higher glenoid concavity is associated with a higher shoulder stability ratio. By showing these results primarily in AP direction the FCSSR showed an increase of 25% compared to BSSR. In SI direction, the same tendency could be shown with an increase of 7.7%, however, not being statistically significant. The comparison between BSSR and OSSR showed only low difference without being statistically different in both directions.

In consequence, by finding significant differences between BSSR and FCSSR as well as between OSSR and FCSSSR, but not between BSSR and OSSR, cartilage as a single criterion did not contribute to a higher glenohumeral SR in these image‐based measurements. The labrum seems to have the major relevance in increasing glenoid concavity and therefore glenohumeral SR.

However, Souleiman et al. could already prove that cartilage contributes to glenoid concavity as well and that severity of cartilage loss has a significant effect on shoulder stability [46]. Underlining this thesis, Wermers et al. were able to show in a previously published study that OSSR delivered a significant higher glenoid concavity compared to BSSR [49]. Moreover, regarding median values of BSSR and OSSR the same tendency towards a higher SR could be shown, as well. Therefore, it can be estimated that this tendency could also show significant results by performing measurements with a higher number of specimens.

At least when comparing MRI‐based OSSR and tactile measurement‐based 3D‐OSSR (labrum excised), a strong correlation could be found, which proved MRI to be an accurate tool for analysing glenoid concavity and therefore SR. However, other studies show inverted results with bigger influence of labrum in SI direction. Halder et al. [11] and Lippitt et al. [25] described the averaged influence of labrum with 10%–20% (7% in anterior, 8% in posterior, 12% in superior, 23% in inferior). An explanation for this inverted trend could be the measuring method. While our findings resulted from measurements of static images (CT and MRI), comparable studies performed their measurements by moving the humeral head over the labrum. Measuring the glenoid concavity in static MRI images could lead to a higher concavity due to negative joint pressure and the ‘suction cup effect’, which results in a ‘lay on’ effect of the labrum onto the humeral head [19]. Additionally, regarding the labrum there are anatomical factors, which influence the measured results further. In the inferior region, the labrum is known as being an immobile extension of the articular cartilage. In contrast, in the anterior region it is only loosely attached, which contributes to a compression of the labrum, while the humeral head slips over it [1, 4, 7]. In consequence, this way of measurement will result in a lower stability regarding the AP direction, while stability measurements of labrum out of static images in CT or MRI lead to an understandable overestimation of stability in AP direction. Another factor, which could influence the results, is an incorrect distinction of the labrum, resulting from the fluid transition of labrum into ligamentous structures in the anterior region [21, 40]. Nevertheless, proving the results to be precise, depth measured in MRI‐i was 1.9 mm deeper than in MRI‐e in AP direction, due to the existence of the labrum. The measured depth in MRI‐i was 4.4 mm. In consequence, the labrum made 43% of total depth, which is similar to findings in current literature (48%–57%) [15]. The importance of labral structures on glenohumeral stability could also be shown by Ishikawa et al. [19], who demonstrated that labral tears lead to a decreased stability ratio and a disappearance of the suction cup effect, which could also be shown by Habermeyer et al. [9]. Moreover, Hu et al. could show that a high stability ratio is significantly associated with a good clinical function and a lower incidence of recurrent shoulder instability in a patient cohort with anterior‐inferior shoulder instability undergoing arthroscopic Bankart repair which underlines the importance of labral structures [16, 17]. Regarding correlations between CT and MRI a strong correlation could be found for depth in AP direction (*r* = 0.68). However, no correlations could be found for depth in SI direction as well as for radius in AP and SI direction and for stability ratio in AP and SI direction. Due to these findings, a transfer of CT measurements to MRI measurements or vice versa seems not to be possible. Moreover, by finding only weak correlations between BSSR/OSSR and FCSSR, morphology of labrum seems to be independent of osteochondral glenoid concavity.

## LIMITATIONS

In this study, a relatively small number of specimens (*n* = 10) was investigated, which is necessary to mention as a limitation. Moreover, specimens were of high age, which could have a relevance for anatomical integrity. To minimise this risk, all specimens were inspected by an experienced surgeon for any pathologies. Moreover, when performing measurements of stability ratio in different imaging modalities, labrum and cartilage are regarded as static, noncompressible soft tissue, which can only give an approach to real glenohumeral stability. Physiologically, cartilage and labrum are under dynamic influence and compression of the humeral head. Besides, focusing on the influence of bony, cartilaginous and labral glenoid concavity and the influence on glenohumeral stability, inclination and version of the glenoid were not analysed in this study, but could have an influence in a biomechanical setting or regarding the in vivo glenohumeral joint [14, 24, 33, 42]. For future research, it would be of interest to prove, if a correlation between FCSSR and radius of the humeral head exists. In consequence, it could be identified, if the labrum itself is a factor for glenoid concavity or if labral concavity is a result of the labrum laying onto the humeral head due to negative joint pressure and the ‘suction cup effect’ [19]. Further investigations with a higher number of specimens should be performed to validate our findings. Moreover, a transfer to clinic would be of high interest, comparing measurements of CT and MRI with clinical outcome of patients.

## CONCLUSION

The influence of cartilage and labrum overall led to a decreased radius and an increased depth, resulting in an increased glenoid concavity and estimated glenohumeral stability. Comparing measurements of FCSSR and BSSR, the influence of labrum and cartilage led to a 25% higher stability ratio in AP direction and a 7.7% higher stability ratio in SI direction. By finding no significant differences when comparing the stability ratio for bony and osteochondral glenoid measurements, the labrum of the glenoid represented the major factor for the increase in shoulder stability ratio. By finding only weak correlations between CT and MRI measurements, a transferability does not seem to be given. Nevertheless, by comparing OSSR and 3D OSSR, MRI could be proven to be accurate for morphometric measurement of glenoid and therefore suitable for evaluating SR.

## AUTHOR CONTRIBUTIONS

Christian D. Schenk was leading author of the article. Sebastian Oenning, Jens Wermers, Marcel Wilken, Julia Sußiek, Arne Riegel, Michael J. Raschke and J. Christoph Katthagen were co‐authors of the article and were accompanying the process of data acquirement and statistical evaluation. Moreover, all of the authors were critically reviewing the paper before submission and had given intellectual input. Arne Riegel was especially supporting the use of the software and therefore the part of measurements. Furthermore J. Christoph Katthagen had given the idea of the study.

## CONFLICT OF INTEREST STATEMENT

The authors declare no conflicts of interest.

## ETHICS STATEMENT

The study was approved by the institutional review board.

## Data Availability

The data that support the findings of this study are available from the Department for Trauma‐, Hand‐ and Reconstructive Surgery, University Clinic Münster upon reasonable request.
